# A new species of *Lygistorrhina* Skuse (Diptera: Sciaroidea: Lygistorrhinidae) from South Africa

**DOI:** 10.3897/BDJ.1.e962

**Published:** 2013-09-16

**Authors:** Vladimir Blagoderov, Laszlo Papp, Heikki Hippa

**Affiliations:** † The Natural History Museum, London, United Kingdom; ‡ Natural History Museum Hungary, Budapest, Hungary; § Swedish Museum of Natural History, Stockholm, Sweden

**Keywords:** Taxonomy, new species, South Africa, Lygistorrhinidae

## Abstract

A new species of *Lygistorrhina* (Diptera, Sciaroidea, Lygistorrhinidae) from South Africa is described and a key for Afrotropical species of the genus is provided.

## Introduction

Lygistorrhinidae is a small family of fungus gnats (Diptera, Sciaroidea) represented by 15 genera and 41 species (http://sciaroidea.info/taxonomy/41555). The genus *Lygistorrhina* includes 21 species which are distributed worldwide in tropical and warm temperate regions. Twelve species of the subgenus *Lygistorrhina (Lygistorrhina*) are known from the Old World (Africa, Eastern Palaearctic, South East Asia, Australasia and Oceania). In addition, an undescribed species of the subgenus was reported from Mexico (Huerta and Ibanez-Bernal 2008). Five Afrotropical species of *Lygistorrhina (Lygistorrhina*) were described from Kenya, Uganda, Côte d'Ivoire, Central African Republic, Gabon, Democratic Republic of Congo and Comoros (Matile 1978, Matile 1990, Matile 1996). In addition, an unnamed species is known from Madagascar (Matile 1996). In this paper we describe a new species of *Lygistorrhina* from South Africa in course of preparation of the chapter on Lygistorrhinidae for the Manual of Afrotropical Diptera.

## Materials and methods

Descriptive terminology follows Söli 1997 and vein nomenclature Shcherbakov et al. 1995. Images of the pinned holotype were taken with an Olympus CP350 compact camera and eyepiece adapter on a stereomicroscope at the Hungarian Museum of Narural History, Budapest (HMNH). Paratypes were originally pinned; one of them was dissected, its wings mounted without media on a microscope slide, and the body was cleared in KOH and stored in glycerol. Details of the paratype were imaged in the Sackler Biodiversity Imaging Lab at the Natural History Museum, London by us of a Canon 450D camera attached to Zeiss Axioskop compound microscope. Additional images and materials are available at the Fungus Gnats Online web-site. All types are held in the HMNH.

## Taxon treatments

### Lygistorrhina austroafricana

Blagoderov, Papp & Hippa, 2013 sp. n.

urn:lsid:zoobank.org:act:FA4CBFA7-1879-43C4-B277-74490A772FDD

http://sciaroidea.info/taxonomy/term/50837

#### Materials

- **Type status:** Holotype. **Occurrence:** catalogNumber: FGO50909; recordedBy: L. Papp & M. Földvári; individualCount: 1; sex: male; **Location:** country: South Africa; stateProvince: Eastern Cape Province; verbatimLocality: Bloukrans Pass, in a side valley; verbatimElevation: 70 m; verbatimLatitude: 33° 57'09.6" S; verbatimLongitude: 23° 37' 59.4" E; **Event:** eventDate: 2007-01-14/16; **Record Level:** institutionCode: HMNH; collectionCode: Diptera
- **Type status:** Paratype. **Occurrence:** catalogNumber: FGO50910; recordedBy: L. Papp & M. Földvári; individualCount: 2; sex: male; **Location:** country: South Africa; stateProvince: Eastern Cape Province; verbatimLocality: Bloukrans Pass, in a side valley; verbatimElevation: 70 m; verbatimLatitude: 33° 57'09.6" S; verbatimLongitude: 23° 37' 59.4" E; **Event:** eventDate: 2007-01-14/16; **Record Level:** institutionCode: HMNH; collectionCode: Diptera
- **Type status:** Paratype. **Occurrence:** catalogNumber: FGO50911; recordedBy: L. Papp & M. Földvári; individualCount: 2; sex: male; **Location:** country: South Africa; stateProvince: Eastern Cape Province; verbatimLocality: Bloukrans Pass, in a side valley; verbatimElevation: 70 m; verbatimLatitude: 33° 57'09.6" S; verbatimLongitude: 23° 37' 59.4" E; **Event:** eventDate: 2007-01-14/16; **Record Level:** institutionCode: HMNH; collectionCode: Diptera

#### Description

Male. Measurements (mm). Head height 0.38, palpi 0.65, proboscis 1.05, antenna 0.9, thorax length 0.63, thorax height 0.71, metepisternum anterior margin 0.15, posterior margin 0.25; coxa 1 0.53; coxa 2 0.47; coxa 3 0.4; wing 1.9.

Colouration. Body, head and antennae entirely dark brownish-grey, almost black, halters yellowish, legs yellowish-brown.

Head (Fig. 7) rounded, vertex slightly convex. Head dichoptic, oval. Ommatidia round, very densely set, equal in size. Interocular setae as long as the diameter of ommatidia. Three ocelli, almost in a straight line, equal in size, set each on its own mound, lateral facing to the sides, medial facing forward. Antenna (Fig. 4) with scape 1.3x shorter than wide, with ventral setae, pedicel spherical, with whorl of setae apically, flagellum 14-segmented, moniliform, flagellomeres with length 1.5x the width, covered with setae 0.5x the width of flagellomeres, flagellomeres 1–11 with a few slightly longer dorsal setae. Bases of setae on flagellomeres form round depressions. Circular bases of flagellomere setae connected with folds forming polygonal pattern. Face as long as wide, with ventral and lateral setae. Clypeus rounded at apex, its length 1.5x the width. Palpi long, 0.6x the length of proboscis, tapering, with a single row of setae in apical 2/3. Proboscis evenly curved caudally, its length 2x the length of fore coxa. Lingua of hypopharynx very thin, transparent, tapering, with very thin hair at each side, longest at tip.

**Figure 1.**
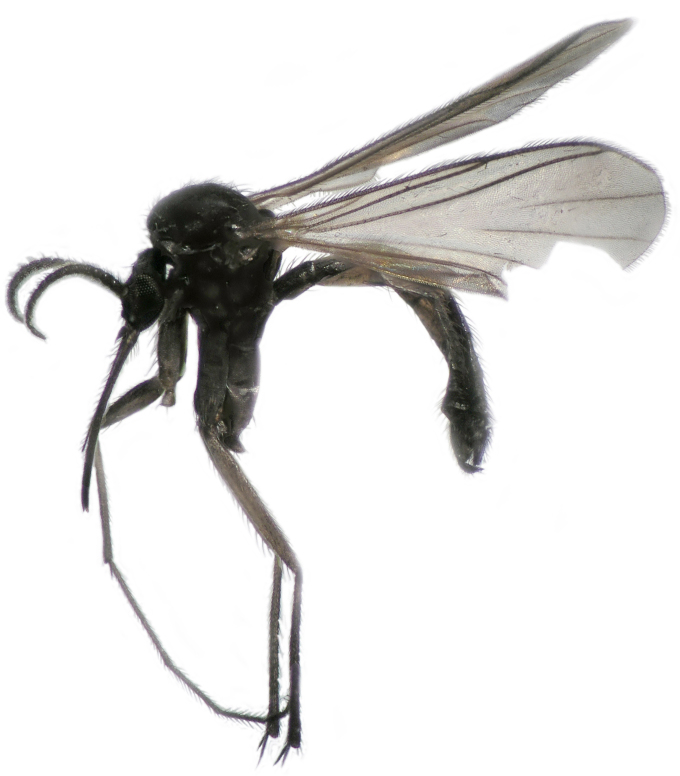
*Lygistorrhina austroafricana*, photograph of holotype (HMNH - Diptera - FGO50909).

**Figure 2.**
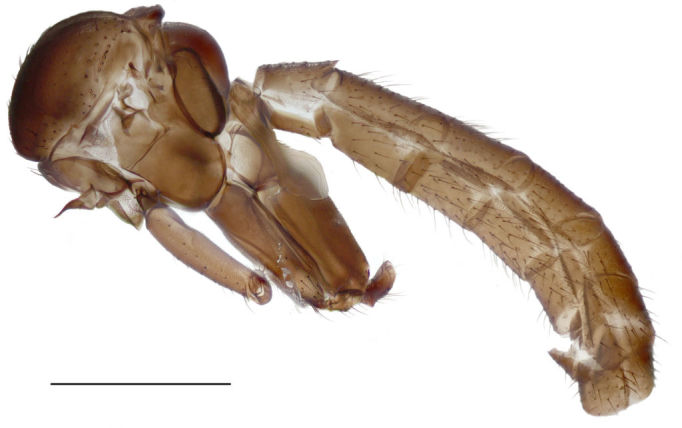
*Lygistorrhina austroafricana*, thorax and abdomen of a paratype (HMNH - Diptera - FGO50910), lateral, photograph. Scale: 0.5 mm.

Thorax, legs, and abdomen uniformly dark brown (Fig. 1). Scutum (Fig. 2) evenly covered with setae of medium length, with longer supraalar setae. Scutellum short, without long setae. Postpronotum apparent, wide. Antepronotum and proepisternum both with 8 setae. Anterior margin of notum well anterior to fore coxa. Ventral margin of preepisternum 2 widely rounded. Metepisternal cleft deep, posterior margin of metepisternum longer than anterior, not extending the level of laterotergite. Laterotergite with a posterior row of 6 long setae. Mediotergite very convex.

**Figure 3.**
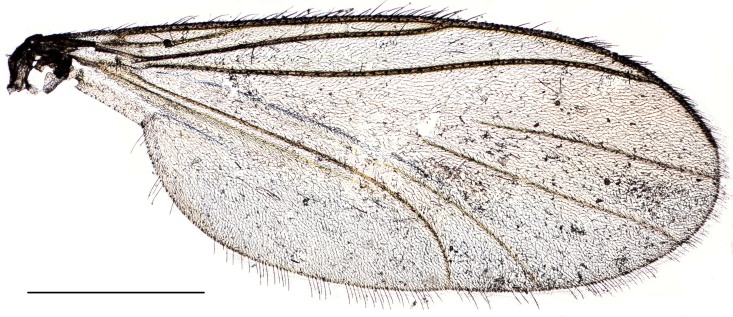
*Lygistorrhina austroafricana*, wing of a paratype (HMNH - Diptera - FGO50910), photograph. Scale: 0.5 mm.

**Figure 4.**
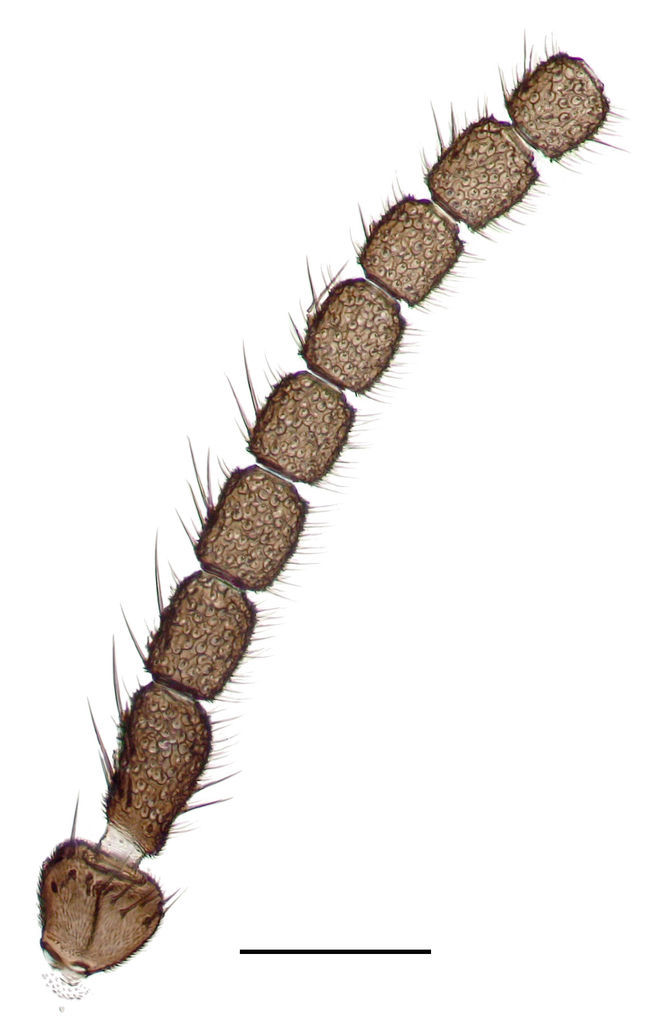
*Lygistorrhina austroafricana*, scape and basal flagellomeres of a paratype (HMNH - Diptera - FGO50910), photograph. Scale: 0.1 mm.

Wing (Fig. 3) hyaline, moderately wide, costal margin slightly convex. Microtrichia on membrane long (0.02 mm), overlapping. Costa with long setae, ending at 3/4 distance between R_5_ and M_1_. Sc ending at C. R_1_ and R_5_ slightly sinusoid, setose dorsally and ventrally. Crossveins *r-m* and *tb* weak but distinct. M_1_ and M_2_ straight, the base of their fork is reduced, M_2_ begins more proximally than M_1_. M_3+4_ and CuA evenly curved caudally, slightly diverging.

Legs (Fig. 5). Fore coxa is the longest, mid coxa a little shorter. Tibial and tarsal setae not in rows. Number of tibial spurs on fore, mid and hind tibia 1:2:2, fore tibia tibial spur short, ca. 1.5x the tibia diameter, mid and hind tibial spurs with inner spur longer, 1.7x and 1.4x the outer, respectively. Hind tibia expanded apically. Fore and mid tarsal claws blunt, curved, with a very small incision at apex and setiform curved basal process as long as the claw. Hind tibia claw pointed, almost straight, with shorter basal process.

Terminalia (Figs 6, 8). Tergite 9 (Figs 6c, d, 8) roughly arrow-shaped with rounded apex, length 0.17 mm, width 0.15 mm, with long scattered setae and a dense patch of short setae at apex. Apodeme of tergite 9 with short, narrow stalk, a little wider that ½ width of the tergite, with two strong semicircular anterior arms. Gonocoxites fused ventrally, with a deep incision, almost reaching the base of synsclerite (Fig. 6b). Synsclerite length 0.2 mm, width 0.26 mm. Gonostyli simple, length 0.13 mm, with a dorsoapical scoop-shaped tooth. Long flagellate setae on mediodorsal edge of gonostylus slightly shorter than gonostylus.

Female. Unknown.

#### Diagnosis

The species differs from all Afrotropical species of *Lygistorrhina* in being smaller (wing length <2 mm), uniformly coloured very dark brownish-grey to black, and having shorter proboscis, which is at most 2x the length of coxa 1. *Lygistorrhina austroafricana* is most similar to *Lygistorrhina edwardsina* Grimaldi & Blagoderov, 2001 (Grimaldi and Blagoderov 2001), but differs in having a wider wing (length/width ratio 2.4 vs 2.7 in *Lygistorrhina edwardsina*) with shorter Sc (0.24x the wing length vs 0.33x) and shorter and wider tergite 9, with stronger anterior arms of apodeme. *Lygistorrhina magna* Matile, 1996 (Matile 1996) also has a uniformly dark coloured body and wide apodeme of tergite 9, but it is a much larger fly (wing length 4.8 mm), and tergite 9 apodeme with stem wide and anterior arms not developed.

#### Etymology

The specific epithet is an adjective in reference to the place of origin of the specimens.

#### Distribution

South Africa: Eastern Cape.

## Identification Keys

### Key to Afrotropical *Lygistorrhina*

**Table:** 

1	Wings with distinct brown spots	2
–	Wings hyaline or smoky, without distinct spots; antennae monochromatic, brown or yellow, at least the first four flagellomeres significantly longer than wide	3
2	Antennae flagellomeres 1–4 brown, 5 orange, 6 bright yellow, 7–11 brown, 12 bright yellow, 5–13-14 light brown; flagellomeres 1–4 twice as long as wide, 5–12 – approximately as long as wide. Gonostili slender, with apical tooth length twice its width. Côte d'Ivoire	*Lygistorrhina hamoni* Matile, 1996
–	Antennae yellow, with flagellomeres 1–3, 7–11 and 14 brown; flagellomeres with length equal to width, except the last two, which are significantly longer than wide. Gonostyli wide, with apical tooth as long as wide. Gabon, Central African Republic	*Lygistorrhina legrandi* Matile, 1990
3	Wings uniformly brownish	4
–	Wings hyaline or very faintly smoky at the apex	5
4	Antennae up to twice the length of the thorax and head together; proboscis yellow; subcosta short obliterated at apex, femora and tibiae brownish yellow, abdominal segments brown with yellowish white edges. Grande Comore	*Lygistorrhina nassreddinei* Matile, 1979
–	Antennae not longer than head and thorax together; proboscis brown; subcosta longer; ending on costa, femur and tibia dark brown; abdomen uniformly brown. Zaire	*Lygistorrhina magna* Matile, 1990
5	Antennae entirely dark	6
–	Antennae entirely yellow; wings hyaline; tibial spurs II equal and shorter than apical width of tibia; internal tibial spur III twice the width of tibia apex. Madagascar	Lygistorrhina sp. (Matile, 1990: 369)
6	Wings very weakly smoky at apex; thorax and abdomen brown to dark brown; proboscis 4x the length of coxa I; tergite 9 elongated, its apodeme thin, with narrow stem; internal tibial spur III 3x the width of tibia apex. Uganda, Kenya, Tanzania, Zaire	*Lygistorrhina edwardsina* Matile, 1990
–	Wings hyaline; body very dark brown, almost black, proboscis ~2x the length of coxa I; tergite 9 of male roughly rounded, its apodeme very wide and thick; internal tibial spur III 2.4x the width of tibia apex. South Africa	*Lygistorrhina austroafricana* sp. n.

(based on Matile 1990, Matile 1996)

## Supplementary Material

XML Treatment for Lygistorrhina austroafricana

## Figures and Tables

**Figure 5a.**
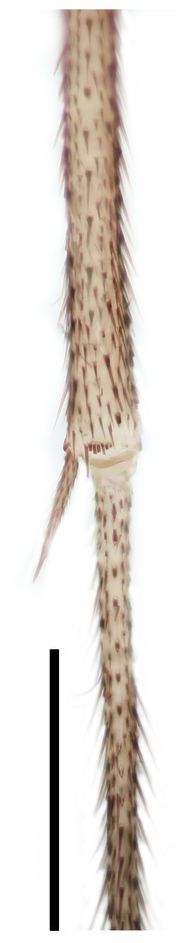
tip of fore tibia

**Figure 5b.**
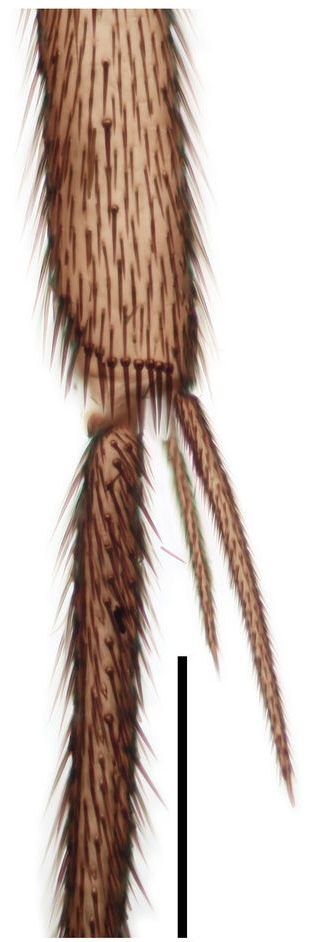
tip of mid tibia

**Figure 5c.**
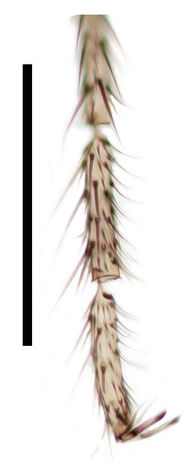
apical tarsomeres of fore tarsus

**Figure 5d.**
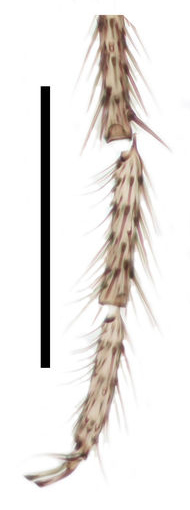
apical tarsomeres of mid tarsus

**Figure 6a.**
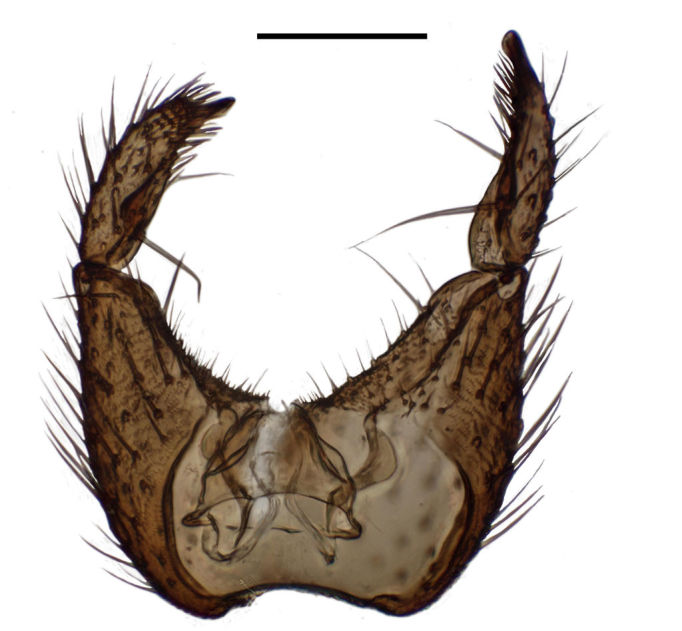
gonocoxites and gonostyli, dorsal view. Scale: 0.1 mm.

**Figure 6b.**
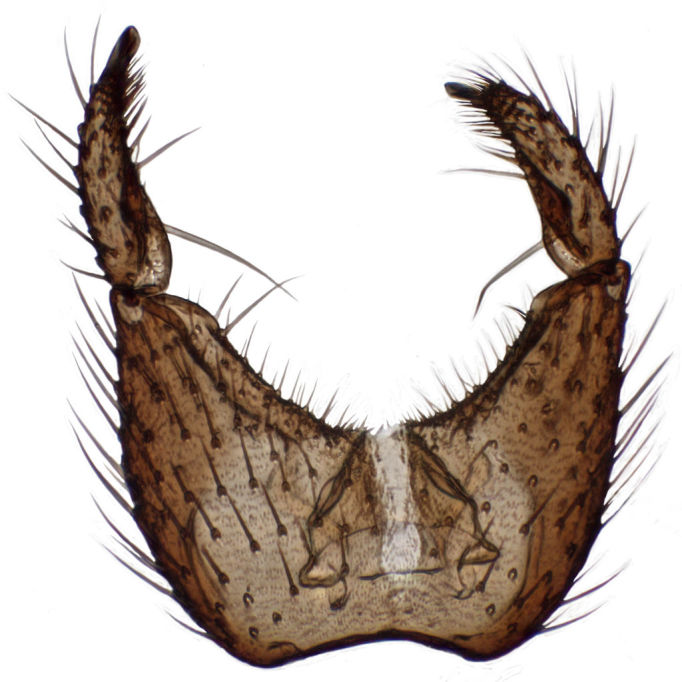
gonocoxites and gonostyli, ventral view

**Figure 6c.**
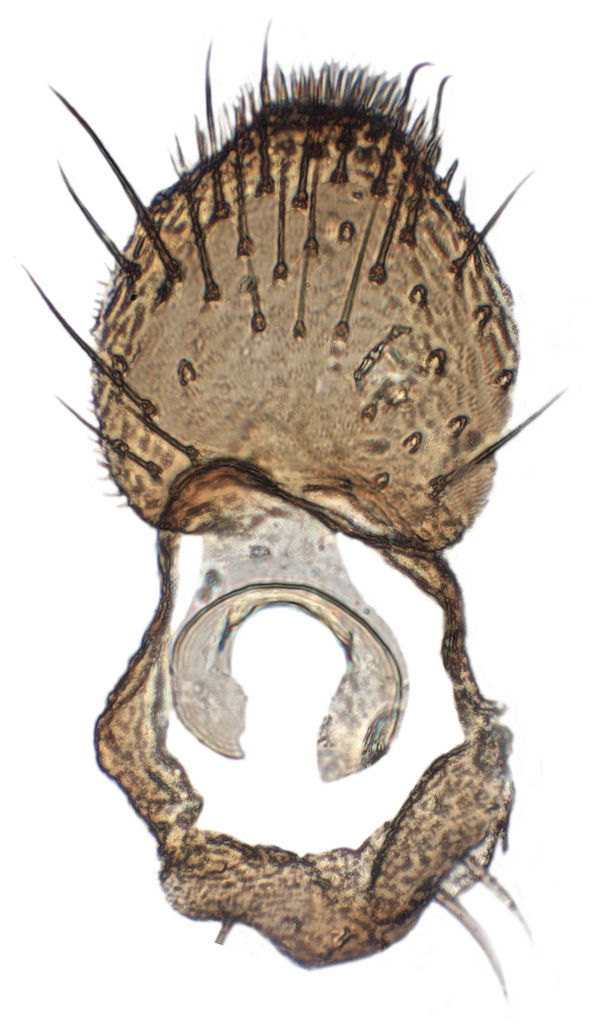
Tergite 9, dorsal view

**Figure 6d.**
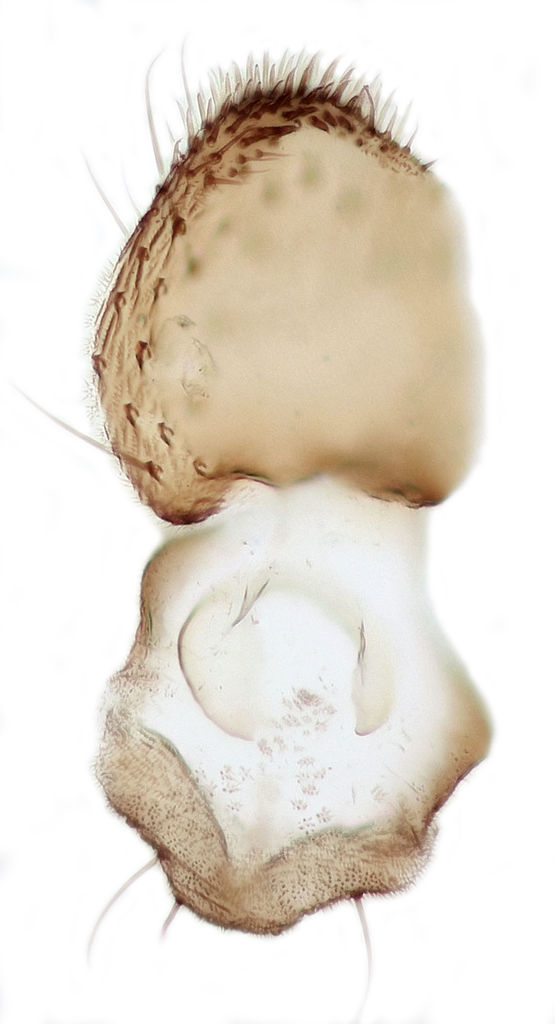
Tergite 9, ventral view

**Figure 7a.**
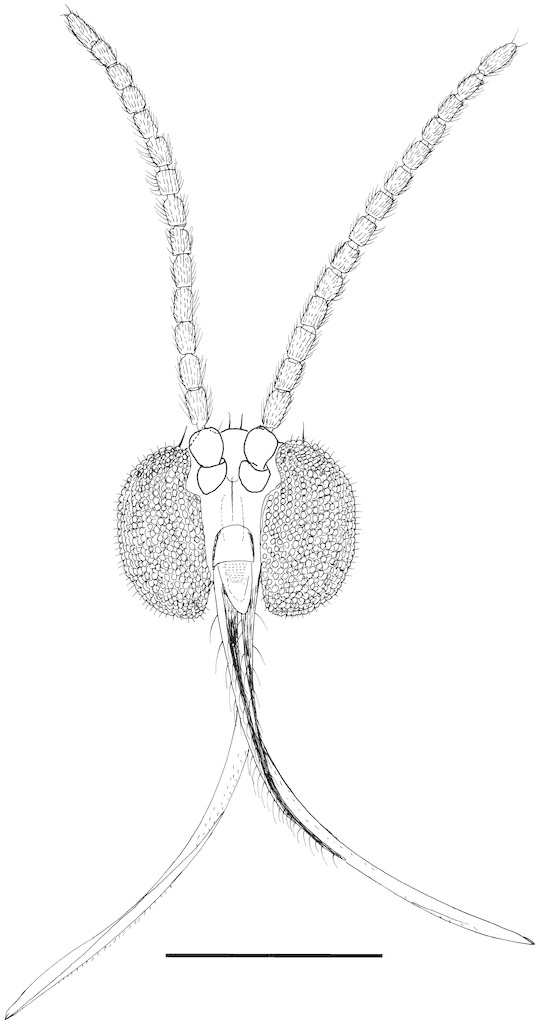
frontal view

**Figure 7b.**
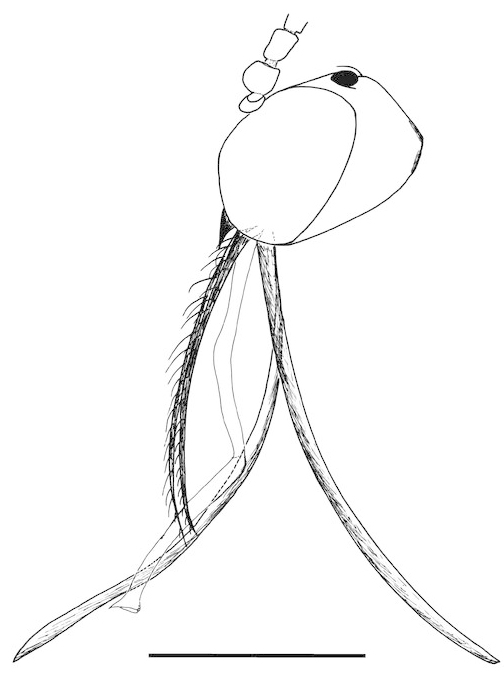
lateral view

**Figure 8a.**
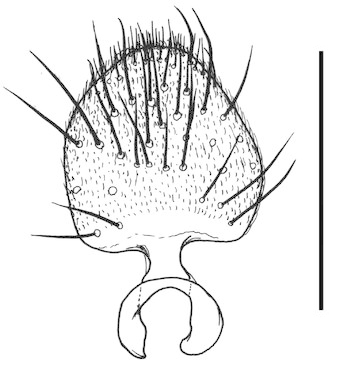
Tergite 9, dorsal view

**Figure 8b.**
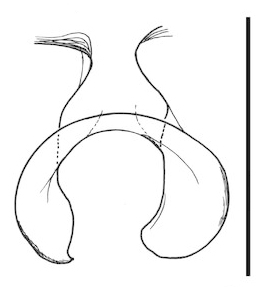
apodeme of tergite 9, dorsal view

**Figure 8c.**
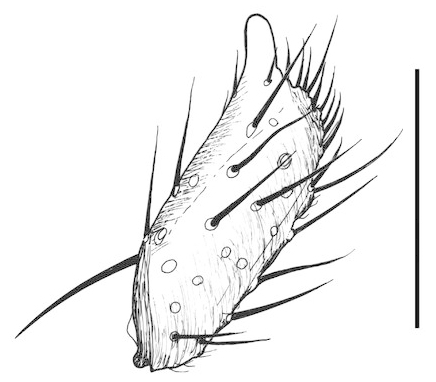
gonostylus, dorsal view

**Figure 8d.**
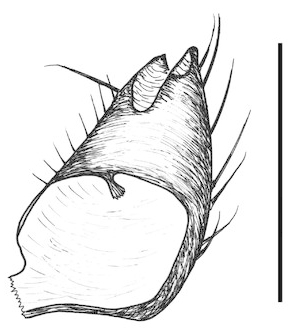
gonocoxite, posterior view from base, dorsal surface to right

**Figure 8e.**
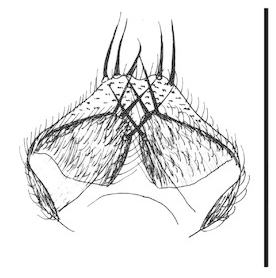
cerci

**Figure 8f.**
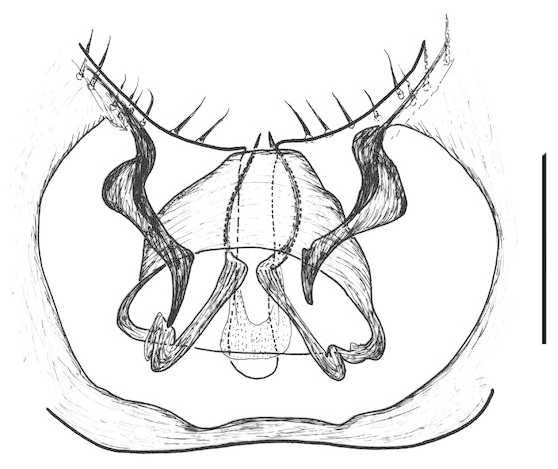
aedeagal complex
